# Poly(rC)-binding proteins as pleiotropic regulators in hematopoiesis and hematological malignancy

**DOI:** 10.3389/fonc.2022.1045797

**Published:** 2022-11-14

**Authors:** Huijuan Zhao, Ziqing Wei, Guomin Shen, Yixiang Chen, Xueqin Hao, Sanqiang Li, Rong Wang

**Affiliations:** ^1^ Henan International Joint Laboratory of Thrombosis and Hemostasis, Henan University of Science and Technology, Luoyang, China; ^2^ Basic Medical College, Henan University of Science and Technology, Luoyang, China; ^3^ Department of Neurology, The First Affiliated Hospital of Zhengzhou University, Zhengzhou University, Zhengzhou, China; ^4^ Department of Clinical Laboratory, Henan Provincial People’s Hospital, People’s Hospital of Zhengzhou University, Zhengzhou, China

**Keywords:** poly(rC)-binding proteins, hematopoiesis, hematological malignancy, mRNA stability, alternative splicing

## Abstract

Poly(rC)-binding proteins (PCBPs), a defined subfamily of RNA binding proteins, are characterized by their high affinity and sequence-specific interaction with poly-cytosine (poly-C). The PCBP family comprises five members, including hnRNP K and PCBP1-4. These proteins share a relatively similar structure motif, with triple hnRNP K homology (KH) domains responsible for recognizing and combining C-rich regions of mRNA and single- and double-stranded DNA. Numerous studies have indicated that PCBPs play a prominent role in hematopoietic cell growth, differentiation, and tumorigenesis at multiple levels of regulation. Herein, we summarized the currently available literature regarding the structural and functional divergence of various PCBP family members. Furthermore, we focused on their roles in normal hematopoiesis, particularly in erythropoiesis. More importantly, we also discussed and highlighted their involvement in carcinogenesis, including leukemia and lymphoma, aiming to clarify the pleiotropic roles and molecular mechanisms in the hematopoietic compartment.

## 1 Introduction

Hematopoiesis is a process wherein hematopoietic stem/progenitor cells undergo cell commitment and terminal differentiation into various blood cell types. It is coordinated and regulated by versatile regulatory factors, including transcription factors, non-coding RNAs, and heterogeneous nuclear ribonucleoproteins (hnRNPs), from direct transcriptional to post-translational control. hnRNPs represent a superfamily of RNA binding proteins (RBPs) interacting with pre-mRNA *via* specific RNA-binding domains. They form complexes to participate in mRNA transport and stabilization, gene alternative splicing, translational enhancement, repression, and chromatin remodeling, thereby controlling the expressions of encoded proteins (1). Prior research has successfully identified approximately 20 protein family members in Homo sapiens by UV cross-linking. They are named hnRNPA1-U alphabetically, and their molecular weights range from 34 to 120 kDa (2). Poly(rC)-binding protein (PCBP) is a subfamily of hnRNPs characterized by high affinity for and sequence-specific interaction with polycytosine, poly(C) (3).

The PCBP family in mammalian cells comprises five protein members divided into two subsets: heterogeneous nuclear ribonucleoprotein K(hnRNP K) (4) and hnRNP E, also named PCBP, or the alpha-complex proteins (α-CP). The four proteins of hnRNP E1-4, also named PCBP1-4, or α-CP 1-4 are also included in the subsets (5, 6). All family members share common structural motifs and triple hnRNP K homology (KH) domains, providing a functional basis for C-rich regions for RNA, ssDNA, and dsDNA binding (5). PCBPs are abundantly expressed and are estimated to constitute approximately 0.5% of the total proteins in mammalian cells (7). Amongst all the PCBPs, hnRNP K, PCBP1, and PCBP2 are ubiquitously expressed in various cell types throughout embryonic development. They are highly expressed in the bone marrow, lungs, liver, kidneys, colon, and other normal tissues or multiple cancer types (8, 9). The inevitable roles of hnRNP K, PCBP1, and PCBP2 in early embryonic development were confirmed by the death of embryonic mice during homozygous gene knockout. Biallelic knockout hnRNP K mouse embryos either fail to form or die *in utero* before E13.5 (10). Mutant mouse embryos homozygous for *Pcbp1* die early during the peri-implantation (E4.5–8.5) stage. Developing mutant Pcbp2 homozygotes proceed normally until E12.5–E13.5 owing to a significant loss in viability associated with combined cardiovascular and hematopoietic anomalies (11, 12). PCBP3 and PCBP4 exhibit limited levels and expression patterns, and little are known about their precise cellular functions.

During the past few years, numerous studies have shown that PCBP family members, especially hnRNP K, PCBP1, and PCBP2, function as modulators in hematopoiesis through multiple-level molecular mechanisms. These include nucleic acid metabolisms such as pre-mRNA alternative splicing, specific mRNA stabilization, transcriptional and translational regulation, or critical iron chaperones delivering iron to target proteins. Considering their functional diversity and complexity, the primary role of PCBPs in regulating gene expression has gained interest in hematopoietic malignancies, such as leukemia and lymphoma. This review summarizes the existing knowledge regarding the structural and functional divergence of various PCBP family members. More importantly, we highlight their roles in normal hematopoiesis, especially erythropoiesis, as well as their involvement in carcinogenesis, including leukemia and lymphoma, over the past decade. Thus, we aim to elucidate the pleiotropic roles and molecular mechanisms of PCBPs in the hematopoietic compartment.

## 2 Structure characteristics and post-translational modifications of PCBPs

### 2.1 Structural characteristics of PCBPs

**Figure 1:**
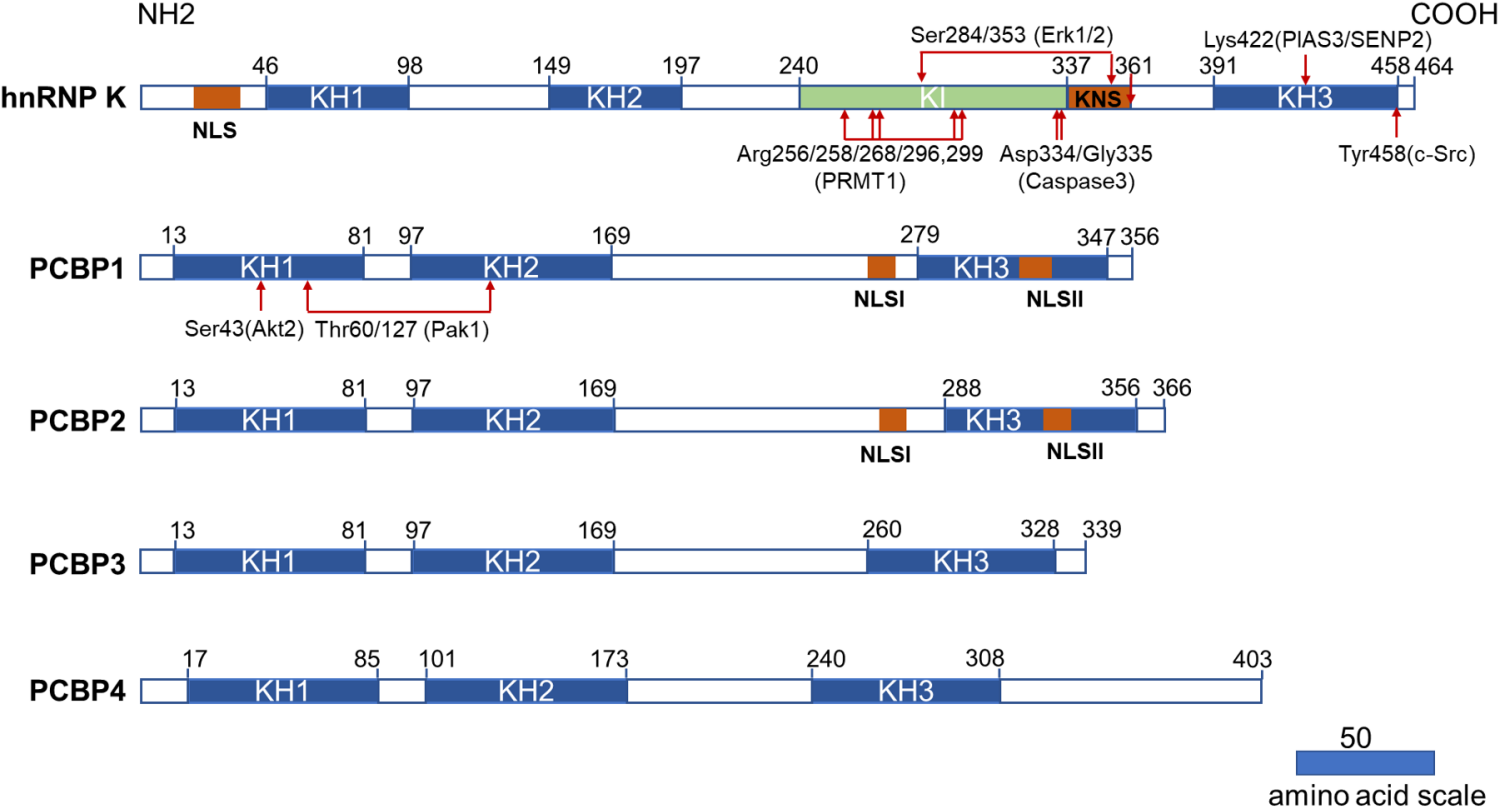
Structural characteristics of PCBPs and their post-translational modifications related to the hematopoietic compartment. All PCBPs consist of triple KH domains. hnRNP K, PCBP1, and PCBP2 have two NLS sequences separately (NLS and KNS in hnRNP K while NLSI and NLSII in PCBP1 and PCBP2). hnRNP K has a unique KI domain responsible for interacting with various proteins. The post-translational modifications and modifying enzymes related to the hematopoietic compartment are all revealed here. KH domain: hnRNP K homology domain; NLS: nuclear localization signal; KI: K-protein-interactive; KNS: hnRNP K nuclear shuttling; PRMT1: protein arginine methyl transferase 1; PIAS3: protein inhibitor of activated STAT3; SENP2: SUMO specific peptidase 2.

All PCBP family members primarily comprise triple hnRNP K homology (KH) domains (KH1, KH2, and KH3) that recognize and combine C-rich regions of RNA, ssDNA, and dsDNA. The KH domain was initially found in hnRNP K as triple repeats (13) and was present in various nucleic acid-binding proteins, including PCBP1–4 (14, 15). Each KH domain comprises 65–70 amino acids and is characterized by a three-stranded antiparallel β-sheet packed against three α-helices (β1-α1-α2-β2-β3-α3). Every KH domain contains a conserved “GXXG” loop linking two α-helices (α1-α2) in the KH core, which specifically binds to poly(C) sequences in both RNA and ssDNA. Protein-nucleic acid interactions are mediated by hydrogen bonding, electrostatic interactions, van der Waals contacts, and shape complementarities (3). The two N-terminal KH domains of PCBPs are closely spaced, whereas the C-terminal is separated by a linker of variable length **(** **Figure 1** **)**. PCBP family members are well-conserved, with the corresponding KH domains sharing a higher degree of homology than the KH domains within each protein (14). The KH domain sequences of PCBP1 and PCBP2 share the highest identity of 93% (14). The primary differences between PCBPs and their isoforms occur in the regions between KH domains, which vary in sequence and length (16). Different KH domains possess different nucleic acid-binding specificities. Although hnRNP K activity is mediated by three KH domains, the KH3 domain plays a crucial binding role, as it has the ability to bind to nucleic acids as an isolated domain, albeit with a lower affinity than that of the full-length protein (17–19). In PCBP1, the KH1 domain forms the most stable interactions with RNA and DNA. KH3 domain binds with intermediate affinity, while the KH2 domain only interacts detectibly with DNA (16, 19–21). KH1 and KH3 domains of PCBP2 can bind to a 7-nt DNA sequence (5’-AACCCTA-3’) and its RNA equivalent. In addition, the crystal structure indicates that PCBP2 dimerizes with KH1-KH1 interaction on exposed surfaces opposite the nucleotide-binding sites. Moreover, KH1 interacts with KH2, similar to KH1–KH1 interaction, whereas KH3 does not dimerize but interacts with the same nucleotide sequence (22–24).

PCBPs also carry a nuclear localization signal (NLS) sequence that mediates transport from the cytoplasm to the nucleus, dictating differential subcellular localization of PCBPs **(** **Figure 1** **)**. Two functionally independent NLS exist in both PCBP1 and PCBP2. A nine amino-acid segment of NLS I is mapped between KH2 and KH3 domains, and NLS II with 12 amino acids are localized in the KH3 domain (25). PCBP1 and PCBP2 are predominantly located in the nucleus, with specific enrichment of PCBP1 in the nuclear speckles. Neither NLS is present in PCBP3 or PCBP4, as both are restricted to the cytoplasm (25). Therefore, they are not regarded as meaningful hnRNPs (1). hnRNP K has a conventional N-terminal bipartite NLS and a nuclear shuttling domain (KNS). Another identified type of nuclear export signal mediates bi-directional transport across the nuclear envelopes (26). The localization of hnRNP K, PCBP1 and PCBP2 in the nucleus and cytoplasm lays the foundation for their multiple roles in transcriptional, posttranscriptional, and translational processes.

In addition to the KH domain, hnRNP K also comprises a unique K-protein-interactive (KI) domain located between KH2 and KH3 domains, which is absent in other PCBPs (5). The KI domain is a docking site that interacts with hnRNP K and various proteins. The domain contains several “RGG” repeats and proline-rich regions responsible for src-homology-3 (SH3)-domain binding, most notably with the SH3 of c-Src family kinases (27). There are several proteins interacting with hnRNP K *via* the KI domain that are involved in gene expression and signal transduction (28). Immunoprecipitation of hnRNP K, followed by mass spectrometry, revealed that hnRNP K is also associated with a host of ribosomal and RNA-processing proteins (29). These studies demonstrated that the KI domain confers hnRNP K with additional functions, such as acting as a docking platform that associates signal transduction pathways with nucleic acid-directed processes. The protein interactome of hnRNP K also contributes to its oncogenicity (29).

### 2.2 Post-translational modifications of PCBPs

Post-translational modifications (PTM) further increase the diversity of PCBPs’ structures and their potential functions, including protein phosphorylation, methylation, and SUMOylation. Generally, PTMs in certain regions can directly influence the subcellular localization of proteins or interact with nucleotides or proteins. hnRNP K possesses the most abundant PTM sites and has recently been comprehensively reviewed by *Xu et al.* (30). Herein we summarized certain PTM of PCBPs related to the hematopoietic compartment.

ERK1/2 dependent phosphorylation of Ser^284/353^ of hnRNP K, located in the KI or KNS region, promotes cytoplasmic accumulation of hnRNP K by affecting nucleocytoplasmic shuttling (31, 32). Cytoplasmic accumulation of hnRNP K subsequently regulates the translation of ALOX15 (33), c-Src (32), and MYC (34). The phosphorylation of Tyr^458^ in KH3 abolishes the mRNA-binding ability and subsequently initiates ALOX15 translation (33). The methylation of specific arginine residues by protein arginine methyl transferase 1 (PRMT1) is another crucial type of PTM for hnRNP K that regulates its interaction with other proteins. Mass spectrometry analysis identified five major methylarginines (Arg256, Arg258, Arg268, Arg296, and Arg299) in the “RGG” motif of hnRNP K (35). The asymmetric dimethylation of hnRNP K during erythroid differentiation at these five arginine residues augments its interaction with RPS19. It suppresses the interaction of its proline-rich domains with the SH3 domain of c-Src, subsequently inhibiting c-Src mediated hnRNP K phosphorylation at Tyr^458^ (36). Furthermore, methylation can strengthen the nuclear localization of hnRNP K (35). A smaller inactive hnRNP K product mediated by caspase 3 at Asp^334^ and Gly^335^ loses its mRNA-binding activity conferred by the KH3 domain upon terminal erythroid differentiation (33, 37). SUMOylation plays an important role in several cellular processes. PIAS3/SENP2 (protein inhibitor of activated STAT3, SUMO-specific peptidase 2) mediates SUMOylation and desumoylation of hnRNPK and controls its stability and interaction with p53, thus leading to cell cycle arrest and recovery (38). SUMOylated hnRNP K at Lys^422^ also positively regulates MYC expression at the translational level, contributing to Burkitt’s lymphoma cell proliferation (39).

PCBP1 and PCBP2 have less diversity in their PTMs. Phosphorylation of PCBP1 and PCBP2 results in a decreased poly(C)-binding activity (6). PCBP1 phosphorylation at Thr^60/127^ by p21-activated kinase 1 (Pak1) leads to its nuclear localization and reduces its binding affinity to mRNA (40). TGF-β-activated Akt2 phosphorylates Ser^43^ of PCBP1, causing PCBP1 release from target mRNAs (41).

## 3 Roles of PCBPs in hematopoiesis

### 3.1 PCBPs in normal erythroid differentiation and function

**Figure 2:**
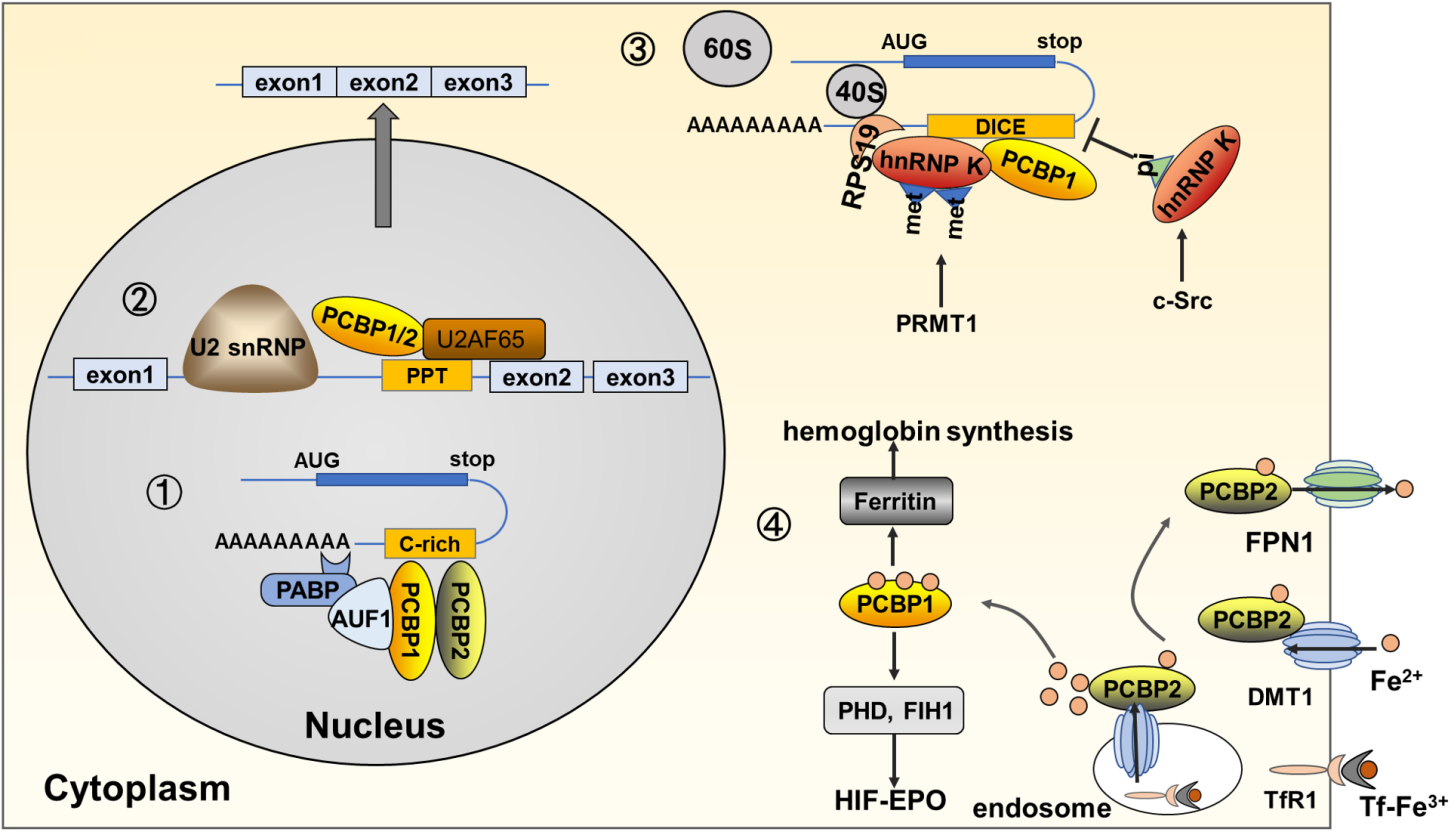
Functions and molecular mechanisms of PCBPs in erythropoiesis. (1) PCBPs bind directly to pre-mRNA 3’UTR and partner with other proteins, such as AUF1, and PABP, to regulate the stability of target mRNAs, such as α/β-globin and EPO. (2) PCBPs bind to the PPT region located 5’ to the PCBP-enhanced exonic segment, together with other spliceosome components, including U2AF65, and U2 snRNP, to induce the alternative splicing of mRNAs, such as protein 4.1R and RUNX1. (3) PCBPs inhibit mRNA translation *via* binding to 3’UTR, precluding 80S ribosome assembly. For example, hnRNP K binds to DICE in ALOX15 mRNA, interacting with RPS19 located at the head of the 40S subunit, inhibiting the 60S subunit from joining the 40S subunit. Post-translational modifications on hnRNP K influence its binding activity; for example, asymmetric demethylation by PRMT1 augments its interaction with RPS19, while phosphorylation of Tyr 458 in KH3 by c-Src dimmish its DICE-binding activity. Other genes of PCBPs mediated mRNA translation repression genes include c-Src, and NMHC IIA; (4) PCBPs act as iron chaperones regulating the cytosolic iron level and mediating hemoglobin synthesis and iron-dependent enzyme activity. PCBP2 directly mediates ferrous iron uptake and export *via* protein-protein interaction with transmembrane DMT1 and FPN1. PCBP2 also transfers ferrous iron in the cytoplasm endosome. Tf-TfR1mediates ferric iron uptake, and in the formed endosome, deoxidized ferrous iron is transferred by DMT1 to PCBP2. PCBP1 behaves as the main protein to transfer ferrous iron to target molecules *via* protein-protein interaction; for instance, PCBP1 transfers iron to ferritin, mediating iron storage and release for hemoglobin synthesis; PCBP1 transfers iron to PHD and FIH1, iron-dependent proteins regulating HIFα stability.

Erythropoiesis, an important part of hematopoiesis, is a highly coordinated and precisely regulated developmental process. The committed erythroid progenitors, BFU-E and CFU-E from hematopoietic stem cells, undergo terminal differentiation into proerythroblasts and basophilic, polychromatophilic, and orthochromatic erythroblasts. They can enucleate to form reticulocytes, release them into the bloodstream, and mature into erythrocytes. With differentiation and maturation, erythroid precursors show a gradual cell and nuclear size reduction. Cell contents are rapidly and progressively replaced with an increased accumulation of hemoglobin (42). PCBPs have specific relevance to normal erythroid differentiation and function and have received great attention for years. In this section, we have discussed the functional diversity of PCBPs based on the various molecular mechanisms involved in erythroid differentiation **(** **Table 1** and **Figure 2** **)**.

**Table 1:** The roles and underlying mechanisms of hnRNP K, PCBP1, and PCBP2 in erythroid differentiation and function.

Family members	Types	Targets	Positions	Functions	References
PCBP1	mRNA stabilization	α-globin	3’UTR	Complex formation stabilizing mRNA	(43)
		β-globin	3’UTR	Complex formation stabilizing mRNA	(44)
		EPO	3’UTR	Complex formation stabilizing mRNA	(45)
	Alternative splicing	Protein 4.1R	Upstream of the 3’ ss of exon 16	Spliceosome complex A formation and Exon 16 inclusion	(46)
		RUNX1	PPT upstream of Exon 6 splice acceptor site	Exon 6 inclusion	(12)
	Translation	ALOX15	3’UTR DICE	Translational silencing	(47, 48)
	Iron chaperone	Ferritin	Protein-protein interaction	Transferring iron and mediating iron storage	(49, 50)
		PHD2/FIH1	Protein-protein interaction	Activation of enzyme	(51)
PCBP2	mRNA stabilization	α-globin	3’UTR	Complex formation stabilizing mRNA	(43)
		β-globin	3’UTR	Complex formation stabilizing mRNA	(44)
		EPO	3’UTR	Complex formation stabilizing mRNA	(45)
	Alternative splicing	RUNX1	PPT upstream of Exon 6 splice acceptor site	Exon 6 inclusion	(12)
	Translation	ALOX15	3’UTR DICE	Translational silencing	(48)
	Iron chaperone	DMT1	Protein-protein interaction	Mediating iron incorporation	(52)
		FPN1	Protein-protein interaction	Mediating iron export	(53)
hnRNP K	Alternative splicing	RUNX1	PPT upstream of Exon 6 splice acceptor site	Exon 6 skipping	(12)
	Translation	ALOX15	3’UTR DICE	Translational silencing	(32, 33, 36, 37, 47, 54, 55)
		c-Src	3’UTR Src3	Translational silencing	(32)
		NMHC IIA	3’UTR U/CCCC-rich element	Translational silencing	(56)

#### 3.1.1 PCBP1 and PCBP2 regulate mRNA stabilization during erythropoiesis

Globin mRNAs accumulate 95% of total cellular mRNA during terminal erythroid differentiation (57),. The accumulation of globin mRNA and its continuous translation is attributable to its stabilization by an mRNP (α-complex) formed at a CU-rich sequence in the 3’UTR. PCBP1 and PCBP2, as the primary components of α-complex, stabilize α-globin mRNA (43). Within the nucleus, PCBP1 and PCBP2, together with hnRNP D (AUF1) and cytoplasmic poly(A)-binding protein (PABP-C), form an α-complex and bind to a 20-nt CU-rich region in the 3’UTR of α-globin mRNA (5, 43, 57, 58). The binding of PABP to the poly(A) tail protects the transcript from deadenylation and degradation, thereby enhancing mRNA stability (59). These roles of PCBP1 and PCBP2 in α-globin mRNA stabilization encouraged subsequent research on the post-transcriptional regulatory functionality of PCBPs. The mRNA-stabilizing complex, including PCBP1 and PCBP2, also binds to the β-globin mRNA 3’UTR, in which a CU-rich motif of 14-nt was identified as the site of mRNP assembly (44, 60). PCBP1 and PCBP2 are also associated with the putative EPO mRNA stability element in the 3’UTR (45). However, another PCBP family member, hnRNP K, cannot bind effectively to the α-globin 3’ UTR, nor can it form an α-complex linked to the stabilization of several additional mRNAs, such as β-globin and EPO (57).

#### 3.1.2 PCBP1 and PCBP2 mediate the alternative splicing of crucial transcripts

Alternative splicing programs produce multiple transcripts, resulting in diverse transcriptomic potential. During erythropoiesis, thousands of genes, including transcription factors and receptors, undergo alternative splicing to satisfy the physiological needs of differentiating erythroblasts (61).

The mechanism of PCBPs’ participation in alternative splicing could enhance cassette exon splicing by binding to a C-rich subset of polypyrimidine tracts (PPT) splice acceptor sites located 5’ to the PCBP-enhanced exonic segment. PCBP1/2, *via* the interaction with the U2 snRNP complex and canonical polypyrimidine tract binding protein, U2AF65, enhances splice acceptor activity and promotes target exon inclusion (62).

Exon 16 of the 80 kDa erythrocyte 4.1R, which encodes peptides within the spectrin/actin-binding domain, is predominantly skipped in early erythroblasts but is included in late erythroblasts (46, 61). The motif “UUUUCCCCCC”, situated at bp -15 to -24 upstream of the 3’ splice site (ss), is vital for exon 16 splicing. PCBP1 binds to the last three-C regions *via* the KH1 domain, along with TIA1, and promotes the recruitment and stabilization of U2 snRNP to a weak branch point by interaction with RBM39, U2AF65, and SF3b155. Thus, PCBP1 stimulates the spliceosome, complex formation, and subsequent inclusion of exon 16 (46).

PCBP2-null mouse embryos lose viability at mid-gestation (E12.5–E13.5), when they undergo prominent defects in the hematopoietic system (11). Furthermore, the loss of PCBP2 results in selective and pronounced impairment of definitive erythropoiesis (12). RUNX1 is a crucial hematopoietic master transcription factor in definitive erythroid lineage (63). Exon 6 of RUNX1 is evolutionarily conservated and encodes the protein domain between DNA-binding and C-terminal transactivation and autoinhibitory domains (12, 64). A C-rich PPT is remarkably conserved from 5’ to the exon 6 splice acceptor site. PCBP2 specifically binds to PPT cytosine content and enhances cassette exon 6 inclusion, which is required for hematopoietic progenitor cells to establish normal myeloid lineages in the fetal liver (12, 62). In K562, a cell line with erythroblast characteristics, PCBP1 enhances exon 6 inclusion at a less pronounced level, while hnRNP K promotes exon 6 skipping (12).

#### 3.1.3 Translational regulation by hnRNP K and PCBP1 in erythroid maturation

The capacity for mRNA synthesis is lost early in erythroid maturation due to nuclear extrusion. However, products of specific genes are needed during late maturation. Timely expression of specific genes controlled post-transcriptionally by PCBPs can satisfy this special demand during erythroid cell maturation. Enucleated reticulocytes are released into the bloodstream mature until mitochondrial degradation occurs. ALOX15, referred to as reticulocyte 15-lipoxygenaser (r15-LOX), is a key enzyme that initiates mitochondrial degradation during the final stage of reticulocyte maturation by catalyzing the deoxygenation of phospholipids in mitochondrial membranes, thus participating in their breakdown in mature reticulocytes (65, 66). ALOX15 mRNA is abundantly transcribed in the normoblast stages but is translationally silenced until enucleated reticulocytes in the peripheral blood undergo the final steps of maturation. Therefore, ALOX15 activity should be temporally restricted by translational silencing in erythroid precursor cells in the bone marrow and early reticulocyte stages. PCBP family members must strictly regulate this process. ALOX15 mRNA 3’ untranslated region (UTR) bears a CU-rich, tenfold repeated 19-nt sequence element referred to as differentiation control element (DICE), mediating translational silencing. The hnRNP K specifically binds to DICE by KH3, whereas PCBP1 binds by KH1 and KH3 (33). hnRNP K and PCBP1 display translation repressor activity alone, but optimal inhibition occurs when both act simultaneously *in vitro* and *in vivo* (47). PCBP2 can substitute for PCBP1 *in vitro* (48). The DICE-hnRNP complex can specifically inhibit the joining of the ribosomal 60S subunit to the 40S subunit at the initiation codon, AUG. Therefore, silencing is not only cap-dependent but also on an internal ribosomal entry site (IRES)-driven translation by the inhibition of 80S ribosome assembly (47, 58, 59, 67–69).

Previous studies have investigated how the mechanism of connection between the DICE-hnRNP K complex to the translation initiation machinery and interference with 60S subunit joining. hnRNP K interacts with ribosomal protein 19 (RPS19), which is localized at the head of the 40S subunit and extends into its functional center, thus inhibiting the 80S ribosome assembly (54). This process can be augmented by the methylation of hnRNP K. Protein arginine methyltransferase 1 (PRMT1) generates the asymmetric dimethylation of hnRNP K at five arginine residues (R256, R266, R268, R296, and R299) (36), augmenting its interaction with RPS19, and causing structural alterations that interfere with 80S ribosome formation (54).

In mature reticulocytes, the ALOX15 translational silenced state must be terminated by PTMs of hnRNP K. This modification includes the methylation, phosphorylation, and proteolytic cleavage of hnRNP K. During maturation, when the level of PRMT1 declines, methylated hnRNP K is replaced by a nonmethylated protein, abolishing the interaction between hnRNP K and RPS19, enabling 80S ribosome formation (54). The non-receptor tyrosine kinase, c-Src, catalyzes the phosphorylation of Tyr458 in the KH3 of hnRNP K and diminishes its DICE-binding activity (33, 55). This process is inhibited by the PRMT1-dependent arginine methylation state of hnRNP K (36). Interestingly, hnRNP K can also control c-Src expression by binding element Src3 in c-Src mRNA 3’UTR, blocking the 80S ribosome assembly, thus inhibiting its translation during the early maturation stage. In later stages, elevated non-methylated hnRNP K functions as a specific activator of c-Src, and the kinase is synthesized (32). Both c-Src and hnRNP K cooperate through intricate feedback control. During erythroid differentiation, a specific N-terminal cleavage intermediate of hnRNP K lacking the DICE binding domain also appears. Caspase-3 acts as an enzyme that cleaves hnRNP K, specifically at amino acids D334-G335, thus removing the C-terminal hnRNP K KH3 domain and conferring hnRNP K binding of DICE (33, 37). The PTM of hnRNP K guarantees the timely release of ALOX15 mRNA from the silencing complex, activating ALOX protein synthesis during erythroid cell terminal maturation. However, translational modifications of PCBP1 that affect its interaction with DICE have not yet been identified in erythroid cells (58).

hnRNP K also regulates erythroid cell enucleation during terminal red blood cell maturation by inhibiting the expression of non-muscle myosin heavy chain (NMHC) IIA (also known as MYH9), an actin-binding protein involved in intracellular vesicle transport. Activated NMHC IIA functions as part of the contractile actinomyosin ring, which is a crucial structure in enucleation. NMHC IIA mRNA levels are constant during erythroid maturation, whereas protein levels increase, indicating translational regulation of NMHC IIA expression. UV-crosslinking and filter-binding assays demonstrate that KH3 of hnRNP K interacts with a specific U/CCCC-rich NMHC IIA mRNA 3′-UTR sequence element, mediating translational silencing in non-induced K562 cells. Simultaneously, translation inhibition was abolished in K562-induced maturation and hnRNP K-depleted K562 cells. K562 cell-induced model recapitulating enucleation and mitochondria degradation results in hnRNP K depletion (56).

#### 3.1.4 PCBP1 and PCBP2 regulate the cytosolic iron level, mediating hemoglobin synthesis and iron-dependent enzyme activity

PCBP1 and PCBP2 are important iron chaperones that bind iron, deliver it to other proteins, regulate cytosolic iron levels in hemoglobin synthesis, and activate enzymes in erythrocyte development.

Iron is taken up by erythroid precursors, delivered to the mitochondria, and converted into heme for hemoglobin assembly. A precise cellular iron level ensures hemoglobin synthesis and does not harm cells. Ferritin, a cytosolic iron storage protein, regulates the cytosolic iron level. Although PCBP1-4 share iron chaperone activity, only PCBP1, and PCBP2 are co-immunoprecipitated with ferritin in HEK293 cells, and PCBP3 can directly interact with ferritin when exogenously expressed in cells (70, 71). Murine erythroid progenitor cells, G1E-ER4 were induced erythroid differentiation. PCBP1 mediates iron storage by binding directly to iron and delivering it to ferritin *via* a metal-mediated protein-protein interaction. During early differentiation, PCBP1 efficiently transfers iron to ferritin for storage when cellular heme synthesis is slow. During late differentiation, when iron demand increases, the amount of iron directed by PCBP1 into ferritin drops (49, 50, 72). Postnatal deficiency of PCBP1 in mice resulted in microcytic anemia due to impaired iron trafficking through ferritin, reduced heme synthesis, and hemoglobin formation (49). However, PCBP2 exhibits the opposite effect, with undetectable ferritin complexes in the G1E-ER4 cell model. With PCBP2 depletion, iron trafficking to ferritin, hemin, and hemoglobin synthesis is enhanced (49). PCBP3 and PCBP4, expressed at lower levels, are insufficient as iron chaperones mediating iron storage (73).

PCBP2, but not PCBP1, binds to divalent metal transporter 1(DMT1), a transmembrane transporter of ferrous iron, which is also crucial for heme synthesis (74). The KH2 domain of PCBP2 interacts with the N-terminal cytoplasmic region of DMT1, and ferrous iron imported by DMT1 is transferred directly to PCBP2 to satisfy iron incorporation (52). Subsequently, PCBP2 interacts with ferroportin 1 (FPN1), an iron export protein, *via* the KH2 domain and C-terminal cytoplasmic region of FPN1 (53).

In addition, as iron chaperones, PCBP1 and PCBP2 can activate iron-dependent enzymes by transferring ferrous iron *via* protein-protein interaction. Prolyl hydroxylases (PHDs) and asparaginyl hydroxylase (FIH1) can modify the hypoxia-inducible factor α (HIFα) protein for degradation *via* the VHL/proteasome pathway. Depleting PCBP1 or PCBP2 in cells leads to a loss of PHD2 activity and accumulation of active HIF1α for EPO transcription (51), which has been previously reviewed (58).

Notably, the domain and mechanism of PCBP1 or PCBP2 binding to ferrous iron have not been clarified, which warrants further research to supplement the versatile roles of PCBPs.

### 3.2 Other hematopoietic lineages

Unlike the specific and pleiotropic relevance of PCBPs with erythropoiesis, their roles in myeloid and lymphoid cell differentiation are limited. They mostly benefit from studies on hematopoietic disorders such as CML and AML, which will be discussed in the subsequent section. PCBPs regulate myeloid-related genes *via* transcriptional and translational regulation. hnRNP K promotes myeloid differentiation by activating MYC expression *via* 5’ cap-independent translation (58, 75), activating C/EBPα and C/EBPβ *via* specific interaction with their promoters (10, 76), and interacting with PU.1, recruited at the CD11b promoter (77). PCBP2 inhibits myeloid differentiation by binding to C/EBP α mRNA and inhibiting its translation (58, 78, 79).

Besides, PCBPs play a critical role in T cell activation. hnRNP K acts as a downstream target of TCR-activated ERK and plays a role in activating two critical transcription factors, NF-κB and NF-AT, in T-cell activation, thus promoting IL-2 production (80). PCBP1 recognizes and binds to CU-rich elements in the 3’UTR of GM-CSF and IL2, stabilizing mRNA and promoting proinflammatory cytokine production in CD4+ T helper cells (81). hnRNP K promotes the immune function of leukocytes by binding to ssDNA within the CD43 gene promoter, mediating its repression (82).

Considering the high expression of hnRNP K, PCBP1, and PCBP2 in bone marrow (8) and their pleiotropic nature, further research is required to clarify the precise role of PCBPs in myeloid (granulocytes, monocytes, and macrophages), and lymphoid (B, T, and NK cell) cell differentiation and functionality.

## 4 Roles of PCBPs in hematological malignancies

**Figure 3:**
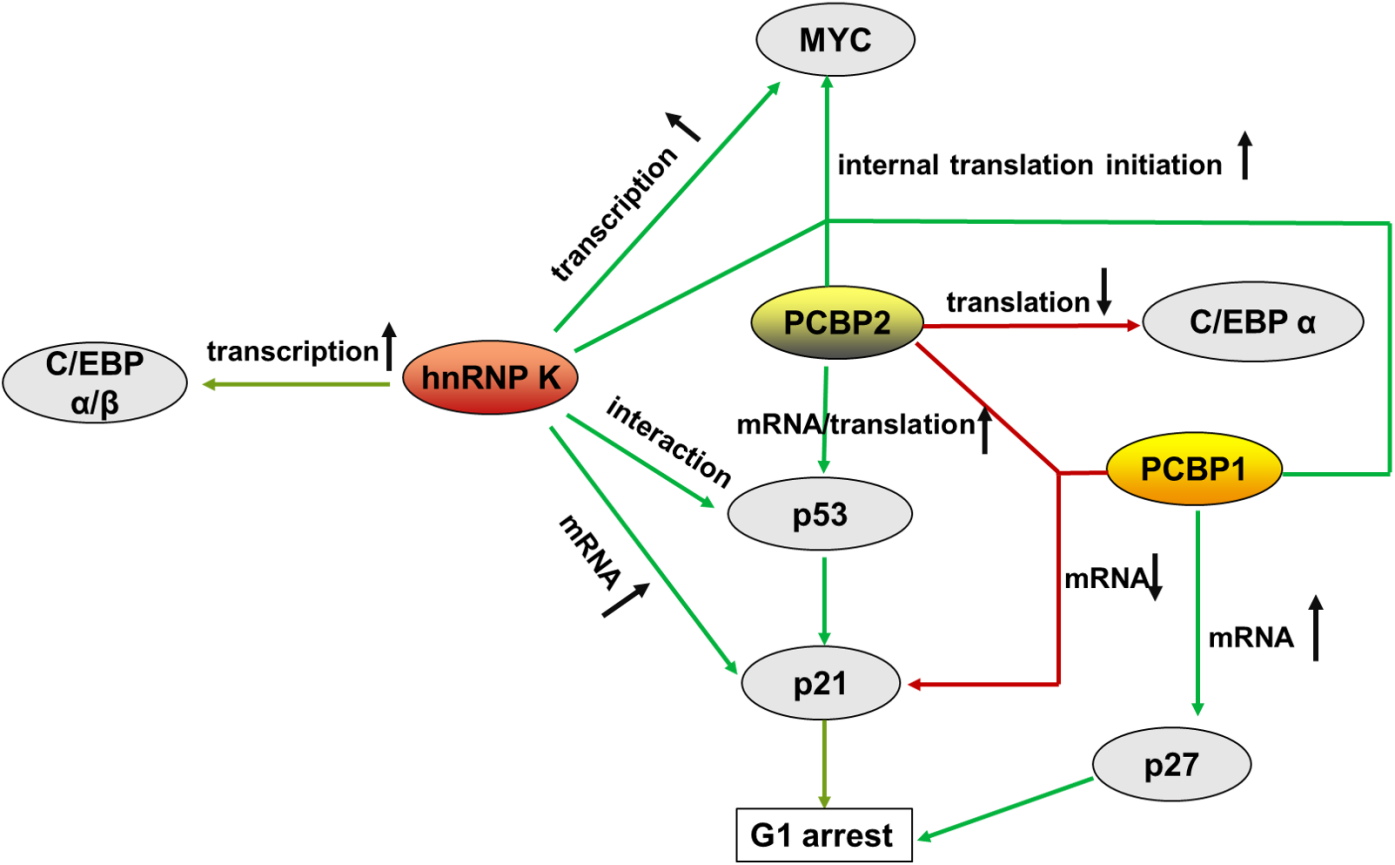
Summary of the main molecules of PCBPs that regulate hematopoietic malignancy, from transcriptional to translational control. represents augment, represents a decrease.

Hematological malignancies are a collection of heterogeneous conditions originating from the bone marrow and lymphatic system and involve three primary groups: leukemia, lymphoma, and plasma cell neoplasms (83). Hematological malignancies share a common characteristic of uncontrolled cell proliferation and are thus involved in a differentiation blockade (84). During this process, the PCBP family members play important roles in versatile biological processes, such as gene activation, repression, and alternative splicing. The relationship between PCBPs and solid cancers has been clarified in detail. For example, hnRNP K is a putative oncogene in several solid malignancies, and its overexpression is associated with poor prognosis (28, 85, 86). PCBP1 acts as a novel tumor suppressor gene in many cancers and is characterized by the downregulation and inhibition of tumor formation and metastasis (87). PCBP2, highly homologous to its family member PCBP1, is expressed in many cancers and is considered an oncogene that promotes tumorigenesis (88). Recent studies have demonstrated that PCBPs have dual oncogenic and tumor-suppressive functions in various contexts. Herein, we summarized the current research on PCBPs in hematological malignancies to identify the roles and mechanisms of PCBPs **(** **Table 2**, **Figure 3** **)**.

**Table 2:** The roles and underlying mechanisms of hnRNP K, PCBP1, and PCBP2 in hematopoietic disorders.

Diseases	Family members	Models	Expression	Roles	Related genes	Effects	Refs
AML	hnRNP K	Patients, hnRNP^+/-^ mice	Low	Tumor suppressor	P21, C/EBPα and β, STAT3	Hematological malignancy with the expansion of myeloid lineages and a significant reduction in survival	(10)
	hnRNP K	Patients, K562 and HL60	High	Oncogene	LC3 I/II	Promoting drug resistance through the regulation of autophagy	(89)
	hnRNP K	Patients,	High	Oncogene	SET	Leukemogenesis and promoting drug resistance	(90)
	PCBP1	Patients	Low	Tumor suppressor	/	/	(91)
	PCBP1 and PCBP2	K562	/	Oncogene	P21	p53-independent induction of p21, with G1 arrest	(92)
CML	hnRNP K	32Dcl3 myeloid precursor, 32D-BCR/ABL	High	Oncogene	MYC	Activation of c-myc translation	(93)
	hnRNP K	patients	High	Oncogene	/	/	(94)
	PCBP2	CML-BC derived EM-3, 32Dcl3 myeloid precursor, 32D-BCR/ABL	High	Oncogene	C/EBPα	Inhibition of C/EBPα translation	(78, 79, 95)
ALL	hnRNP K	patients, Ph+ ALL cell lines (SUP/B15)	High	Oncogene	Beclin1	Enhancing autophagic activity by interaction with the Beclin1 mRNA	(96)
DLBCL	hnRNP K	patients, transgenic mouse model	High	Oncogene	MYC	Activation of c-myc translation, development of lymphomas, with poor clinical outcomes and therapy resistance	(93)
Burkitt lymphoma	hnRNP K	human Burkitt lymphoma cell line (Daudi, CA46)	High	Oncogene	MYC	Activation of c-myc translation	(39)
	PCBP1	patients	/	/	/	Somatic mutations altering PCBP1 function	(97)

In hematological tumorigenesis, two conventional tumorigenesis-related signaling pathways, the oncogenic MYC pathway and the onco-suppressive p53/p21 pathway, are influenced by PCBPs, making it plausible that the same gene can be oncogenic and tumor-suppressive simultaneously.

*MYC* (*c-Myc*) is a proto-oncogene involved in multiple cancers that impacts many biological events, including cell proliferation, differentiation, and programmed cell death, when aberrantly regulated. MYC plays an important role in the maturation and expansion of myeloid and lymphoid cells. Uncontrolled MYC elevated expression is often observed in leukemia and lymphoma (98). PCBPs are complex regulators of MYC that promote its expression in the hematopoietic compartment. hnRNP K transcriptionally promotes MYC expression by interacting with the CT-rich regions in the promoter of the *MYC* gene (99, 100). In addition, hnRNP K, PCBP1, and PCBP2 interact with the MYC IRES located in the 5’UTR, aiding its entry to the ribosome through a cap-independent mechanism for alternatively internal translation initiation (101). A mutated version of the MYC IRES prevalent in multiple myeloma (MM) binds tightly to hnRNP K, thus promoting increased aberrant expression of MYC (101).

The p53/p21 pathway inhibits cell cycle and proliferation by controlling the G1 checkpoint (102). p53 is a potent tumor suppressor and the primary transcriptional regulator of p21. p53/p21 dysregulation is highly correlated with hematopoietic disorders. The highest levels of p53 are strongly associated with the advanced phases of CLL, ALL, CML, and AML *de novo* and relapse cases (103). *TP53* mutations are associated with resistance to chemotherapy (104, 105). p21, a downstream factor of p53, functions as a cyclin-dependent kinase (CDK) inhibitor that directly mediates cell cycle arrest in the G1 phase. It is dysregulated in some cancers (106), including hematopoietic tumors (107, 108). PCBPs regulate p53/p21 expression through multiple mechanisms. hnRNP K interacts with p53 and acts as a cofactor for p53-mediated p21 transcription (109, 110). Moreover, hnRNP K stabilizes p21 mRNA by binding to its 3’UTR (111, 112). PCBP2 enhances p53 mRNA stability by interacting with its 3’ end stimulating its translation *via* specific binding to the 5’-terminal region of p53 mRNA (113). In contrast to PCBPs’ mRNA stabilization, PCBP1 and PCBP2 bind to p21 mRNA and destabilize the transcript in a p53-independent pathway (92).

### 4.1 AML

Acute myeloid leukemia (AML) is one of the most common and aggressive malignancies of the hematopoietic system. hnRNP K haploinsufficiency has been discovered in AML patients, contributing to the disease process (10). Approximately 2% of all AMLs harbors 9q21.32 deletions with one *HNRNPK* allele lost. The *HNRNPK* in the CD34+ cells of these patients was dramatically decreased compared to that in healthy donor samples. Mice embryos with biallelic loss of *Hnrnpk* failed to form or died at E13.5. Haploinsufficient mice (*Hnrnpk* ^+/−)^ mimicking the putative haploinsufficiency observed in AML patients were significantly smaller and had a decreased survival rate. The reduced hnRNP K expression led to the hematological malignancy development with a significant increase in neutrophils, basophilic granulocytes, platelets, and CD11b/LyG6 double-positive cells. The serum levels of interleukin (IL)-3 and IL-6, which promote the proliferation of myeloid cells, were also increased. Additionally, *Hnrnpk^+/-^* mice frequently harbor other hematologic malignancies, including T- and B-cell lymphomas (10). hnRNP K acts as a tumor suppressor in AML by regulating the p53/p21, CEBP, and STAT3 pathways (10). A reduced hnRNP K expression attenuated p21 activation (10). hnRNP K, a p53 cofactor (109, 110), is required for p21 transcription (114). A chromatin immunoprecipitation (ChIP) assay in *Hnrnpk*+/− mice tissues showed that hnRNP K directly interacts with the *p21* gene (10). In addition, hnRNP K was reported to stabilize p21 mRNA by binding to the 3’UTR (111, 112). hnRNP K is required for efficient G1 cell-cycle arrest (110). Furthermore, the reduction of hnRNP K in AML significantly downregulated C/EBPα and C/EBPβ levels (10), by interacting with their promoters (10, 76). C/EBPα p42 isoform, a myeloid tumor suppressor (115), was significantly downregulated in *Hnrnpk* ^+/−^ mice, suggesting that hnRNP K might also interact with the C/EBPα transcript. hnRNP K loss in *Hnrnpk* ^+/−^ mice also significantly activated STAT3 expression, which directly contributed to the differentiation and proliferation defects in the hematological compartment (10).

However, hnRNP K might be an oncogene due to its significant overexpression in the bone marrow of non-remission AML patients and drug-resistant cell lines (89, 90). hnRNP K expression was positively correlated with that of autophagy-related proteins such as microtubule-associated protein light chain 3 I and II (LC3I/II). This indicates that hnRNP K might promote the survival of leukemic cells by influencing autophagy (89). The relationship between hnRNP K and tumorigenesis is difficult to address, primarily because of the number of cellular processes it regulates. However, the studies above revealed that hnRNP K acts mainly as a tumor suppressor in AML (86).

Another family member, PCBP1, also acts as a tumor suppressor in AML. PCBP1 expression was significantly decreased in newly diagnosed AML patients and recovered with complete remission. Clinical feature analyses showed that the white blood cell count was elevated in patients with low PCBP1 expression. Besides, patients with low PCBP1 expression had poor disease-free and overall survival (91). PCBP1 can suppress cell proliferation and carcinogenesis by binding to the 3’-UTR of p27 *via* its KH1 domain to stabilize p27 mRNA and enhance its translation (116). Additionally, PCBP1 inhibits the expression of STAT3, an oncogene frequently overexpressed in cancer, through the interaction with its 5’-UTR (117).

However, PCBP1 may promote tumorigenesis by co-depleting with PCBP2. In K562, an acute myeloid leukemia cell line, the knockdown of PCBP1 or PCBP2 alone did not affect cell growth. In contrast, PCBP1 and PCBP2 co-depletion inhibited cell proliferation and triggered a significant G1 cell cycle arrest, with an induction of p21 protein expression. Interestingly, in contrast to mRNA stabilization, PCBP1 and PCBP2 can bind to and destabilize p21 mRNA *via* a 127‐bp CU‐rich region in the 3′‐UTR. This study highlighted the novel activities of PCBP1 and PCBP2 in tumorigenesis (92). The role of PCBP2 in AML patients has not yet been reported.

### 4.2 CML

Chronic myelogenous leukemia (CML) is a myeloproliferative disorder caused by deregulated tyrosine kinase activity of p210-BCR/ABL oncoprotein, the product of t(9;22) (q34;q11) translocation (118). CML progression from the chronic phase (CML-CP) to the acute and fatal blast crisis (CML-BC) phase impairs the ability of myeloid progenitors to differentiate terminally into mature neutrophils (118).

In CML patients, hnRNP K levels are more abundant in mononuclear cells (MNCs) from CML-BC patients than in CML-CP cells (94). hnRNP K and PCBP2, as oncogenes, contribute to the progression of CML-CP to CML-BC *via* distinct pathways through the activation of MYC mRNA translation and suppression of C/EBP α mRNA translation (58). The constitutive tyrosine kinase BCR/ABL activates downstream MAPK^ERK1/2^, which phosphorylated both hnRNP K (Ser^284/353^) (75) and PCBP2 (Ser^173/189/272^ and Thr^213^) (79), thereby increasing protein stability and cytoplasmic accumulation. In CML-BC progenitors, hnRNP K binds to the IRES of MYC and promotes MYC translation (75), accounting for BML-BC cell proliferation and colony formation (119, 120). PCBP2 binds to C/EBPα, the principal regulator of granulocytic differentiation, *via* a pyrimidine motif (CUCCCCC) in its 5’-UTR that spaces an upstream open reading frame (uORF) (18-nt) from the main ORF (78). Because uORFs are involved in translational control (121), C/EBPα expression is suppressed at the translational level. Reduced C/EBPα expression downregulates GCSF-R transcription, contributing to granulocyte differentiation (64). miRNAs also regulate PCBP2-mediated translational repression of C/EBPα. miR328 competes with C/EBPα mRNA to bind to PCBP2. The loss of miR328 in CML-BC abolished the translation of C/EBPα repression (95).

### 4.3 ALL

hnRNP K overexpression was assessed in Philadelphia chromosome-positive acute lymphoblastic leukemia (Ph(+) ALL) patients and imatinib-resistant ALL cell lines. hnRNP K knockdown increased the imatinib sensitivity of these tumor cells and decreased the *in vivo* tumor burden. hnRNP K enhances autophagic activity in drug-resistant Ph+ ALL cells by iteration with the mRNA of Beclin1, an autophagy-related protein (96).

### 4.4 B-cell lymphoma

Malignant lymphomas constitute a heterogeneous group of neoplasms derived from cells of the immune system (B-, T-, NK-lymphocytes) (83). More specifically, lymphomas are classified as Hodgkin’s and non-Hodgkin’s (HL and NHL), accounting for 10% and 90% of the cases, respectively. Diffuse large B-cell lymphomas (DLBCL) and Burkitt’s lymphoma are two subtypes of NHL and B- cell lymphomas, respectively. DLBCL constitutes nearly half of all NHL cases, making it the most commonly diagnosed lymphoma subtype (122).

In DLBCL patients without MYC genomic alterations, high hnRNP K expression is associated with poor overall survival, progression-free survival, and lack of response to chemotherapy. A transgenic mouse model with hnRNP K overexpression also exhibited lymphomagenesis and a reduced survival rate. Mechanistically, hnRNP K activates MYC expression through posttranscriptional and translational regulation, contributing to DLBCL pathogenesis (29, 93).

Burkitt’s lymphoma is the most common B-cell lymphoma in children. Its genetic hallmark is the translocation t(8;14) (q24;q32) involving the MYC gene and the immunoglobulin heavy chain (IGH) gene or its light chain variants, bringing the MYC oncogene under the influence of immunoglobulin gene enhancers. This translocation deregulates MYC expression, contributing to Burkitt’s lymphoma cell proliferation (84). hnRNP K overexpression was also observed in Burkitt’s lymphoma, whereby SUMOylated hnRNP K at Lys422 was elevated. SUMOylated hnRNP K positively regulates MYC expression at the translational level by increasing the binding activity of MYC 5′ UTR IRES (39).

A next-generation sequencing study identified recurrent somatic mutations in PCBP1 in Burkitt’s lymphomas (3/17, 18%). The mutations predominantly (83%) affect the PCBP1 KH3 domain, either by complete domain loss or amino acid changes. Missense mutations in two cases within NLSI or NLSII may solely lead to decreased PCBP1 expression within the nucleus. These mutations were predicted to alter various functions of PCBP1, including nuclear trafficking and pre-mRNA splicing (97). Notably, mutations in genes encoding other important RNA splicing factors, such as SF3B1 and U2AF1, are currently among the most recurrent genetic abnormalities in patients with myeloid neoplasms and lymphoproliferative disorders (123).

## 5 Concluding remarks

PCBPs, the triple KH domain proteins interaction with C-rich regions of RNA, ssDNA, and dsDNA and exert abundant biological functions of cellular events, including proliferation, differentiation, apoptosis, DNA damage, repair and stress, and immune responses (73). Herein, we summarized the functional divergence of various PCBP family members in normal hematopoiesis and hematopoietic malignancies. Conclusively, PCBPs regulate hematopoietic cell differentiation and cell function by controlling gene expression *via* modulation of mRNA stabilization, alternative splicing, translational repression, and activation, together with other additional factors, such as PABP-C, U2AF65, and RPS19. PCBPs are particularly relevant for erythroid differentiation and maturation. In addition to their involvement in nucleic acid metabolism, PCBPs act as iron chaperones, regulating the cytosolic iron levels for hemoglobin synthesis or activating crucial enzymes. Notably, the specific binding sites of PCBP1 and PCBP2 for ferrous iron are still unclear, and the exact mechanism by which PCBP1 delivers ferrous iron to its target proteins remains inconclusive.

Post-transcriptional regulation is crucial in orchestrating gene regulatory networks in hematopoietic cell growth, differentiation, and tumorigenesis. The roles of other RNA-binding proteins in acute leukemia have recently been fully elucidated (86). Because PCBPs regulate gene expression on multiple levels, such as acting as transcriptional activators, regulators of RNA splicing and translational repressors, their roles in hematopoietic malignancies have gained increasing attention. In the past decade, many studies showed the relevance of PCBP family members, especially hnRNPK, PCBP1, and PCBP2, in AML, ALL, and others. Altered genes have historically been designated as either oncogenes or tumor suppressors. Owing to the pleiotropic nature of the PCBP family, their over- and under-expression can be pathogenic. It is difficult to identify the precise role of PCBPs in a particular type of hematopoietic disorder, as the same family member may exert cogenetic and tumor suppressive effects in different cell contexts or differentiation stages. This dualism is mainly based on PCBPs’ multifunctionality, as they act in many ways in the cytoplasm and nucleus due to their unique localization. In further research, an in-depth study of the interactions between PCBPs and RNAs or proteins should be considered comprehensively. Using Clip-seq, RIP-Seq, or RNA pulldown at the genome-wide level and using protein Co-IP and mass spectrographic analysis at the protein-interactome level, might provide more definite information for understanding the regulatory networks of PCBPs in hematopoietic disorders. Significantly, it may be more plausible to track the involvement of PCBPs in different disease phases with the progression of hematopoietic disorders.

Elucidating the roles of PCBPs in hematopoietic malignancies may provide a valuable therapeutic modality in the future. Certain studies have demonstrated that hnRNPK, PCBP1, and PCBP2 may serve as prognostic biomarkers and potential therapeutic targets. Although no established PCBPs-specific therapies currently exist, elucidating the role of PCBPs in hematopoietic malignancies may provide valuable insights. For instance, *in vivo* study demonstrated that hnRNP K is a crucial regulator of shaping leukemia cell resistance to imatinib (96), partly explaining the mechanism of leukemia cell drug resistance. hnRNP K overexpression in DLBCL patients without MYC genomic alterations renders cells sensitive to BET-bromodomain inhibitors both *in vitro* and in transplantation models. This suggests that BET-bromodomain inhibitors could be an effective therapeutic modality in the treatment of malignancies characterized by hnRNP K overexpression (93).

Besides, the roles of the other two family members, PCBP3 and PCBP4, in the hematopoietic compartment have not yet been revealed. Research on PCBP3 and PCBP4 in other contexts could offer valuable insights. For example, in neuronal cells, PCBP1, PCBP2, and hnRNP K work simultaneously to stimulate the µ-opioid receptor gene, while PCBP3 works as a repressor (124, 125). PCBP4 can inhibit the proliferation and tumorigenesis of lung cancer cells by delaying the cell cycle progression (126).

Conclusively, the functional diversities of PCBP family members involved in the hematopoietic compartment and their pleiotropic molecule mechanisms will be revealed more clearly with further research.

## Author contributions

HZ, LS, RW, and XH contributed to the study design. HZ prepared and wrote the manuscript. ZW, GS and YC critically reviewed and revised the manuscript. All authors contributed to the article and approved the submitted version.
