# Corrigendum: Poly(rC)-binding proteins as pleiotropic regulators in hematopoiesis and hematological malignancy

**DOI:** 10.3389/fonc.2022.1132485

**Published:** 2023-02-14

**Authors:** Huijuan Zhao, Ziqing Wei, Guomin Shen, Yixiang Chen, Xueqin Hao, Sanqiang Li, Rong Wang

**Affiliations:** ^1^ Henan International Joint Laboratory of Thrombosis and Hemostasis, Henan University of Science and Technology, Luoyang, China; ^2^ Basic Medical College, Henan University of Science and Technology, Luoyang, China; ^3^ Department of Neurology, The First Affiliated Hospital of Zhengzhou University, Zhengzhou University, Zhengzhou, China; ^4^ Department of Clinical Laboratory, Henan Provincial People’s Hospital, People’s Hospital of Zhengzhou University, Zhengzhou, China

**Keywords:** poly(rC)-binding proteins, hematopoiesis, hematological malignancy, mRNA stability, alternative splicing

## Incorrect Affiliation

In the published article, there was an error in affiliations 1 and 2. Instead of “^1^Basic Medical College, Henan University of Science and Technology, Luoyang, China, ^2^Henan International Joint Laboratory of Thrombosis and Hemostasis, Henan University of Science and Technology, Luoyang, China”, it should be “^1^Henan International Joint Laboratory of Thrombosis and Hemostasis, Henan University of Science and Technology, Luoyang, China, ^2^Basic Medical College, Henan University of Science and Technology, Luoyang, China”.

The authors apologize for this error and state that this does not change the scientific conclusions of the article in any way. The original article has been updated.

